# The demise of extracorporeal membrane oxygenation in infarct‐related cardiogenic shock

**DOI:** 10.1002/ctm2.1485

**Published:** 2023-12-22

**Authors:** Steffen Desch, Uwe Zeymer, Anne Freund, Holger Thiele

**Affiliations:** ^1^ Department of Internal Medicine/Cardiology Heart Center Leipzig at the University of Leipzig Leipzig Germany; ^2^ Department of Cardiology, Pulmonology, Angiology and Intensive Care Medicine Klinikum Ludwigshafen Ludwigshafen Germany; ^3^ Institut für Herzinfarktforschung Ludwigshafen Germany

Cardiogenic shock in acute myocardial infarction carries a dismal prognosis. In‐hospital or 30‐day mortality reaches 40% to 50%.^1^ Timely treatment of the coronary culprit lesion (usually by means of percutaneous coronary intervention) is the only treatment proven effective in reducing mortality within a randomized controlled trial.^2^, ^3^ It is against this background that advances in treatment are urgently needed.

High expectations have been placed on venoarterial extracorporeal membrane oxygenation (VA‐ECMO) and its use has almost become inflationary in recent years.^4^ Blood is actively pumped into a tubing system outside the body which also incorporates an artificial lung for oxygenation and removal of carbon dioxide and then sent back retrograde in the aorta. The idea is to provide temporary circulatory (and respiratory) support during the critical first days until the failing heart has recovered enough to take over again. However, VA‐ECMO carries serious risks and evidence for an overall clinical benefit from well‐controlled randomized studies has been largely missing.

The recently published multicenter Extracorporeal Life Support in Infarct‐related Cardiogenic Shock (ECLS‐SHOCK) trial is the first adequately powered randomized study to address whether VA‐ECMO impacts survival in infarct‐related cardiogenic shock.^5^ For those who had hoped for progress in treatment, the results were a bitter disappointment. In short, 420 patients with acute myocardial infarction complicated by cardiogenic shock and planned for early revascularization were randomly assigned to receive standard care with or without early VA‐ECMO. There was no significant difference in the primary endpoint of all‐cause death at 30 days (47.8% in the VA‐ECMO group versus 49.0% in the control group, *p* = .81, Figure 1). However, there was a much higher rate of moderate or severe bleeding and peripheral vascular complications with VA‐ECMO. There was no signal for a survival benefit of VA‐ECMO in any of the subgroups analyzed, namely age < 65 or ≥65 years, sex, diabetes, ST‐segment or no ST‐segment elevation myocardial infarction, anterior or non‐anterior myocardial infarction, moderately elevated or high lactate levels, or whether the patient had been resuscitated before hospital admission or not. Subgroup analyses should always be met with a certain degree of caution. With neutral results, low power to detect treatment effects must be taken into consideration. And there is a chance to miss out on subgroups that might be relevant.

**FIGURE 1:**
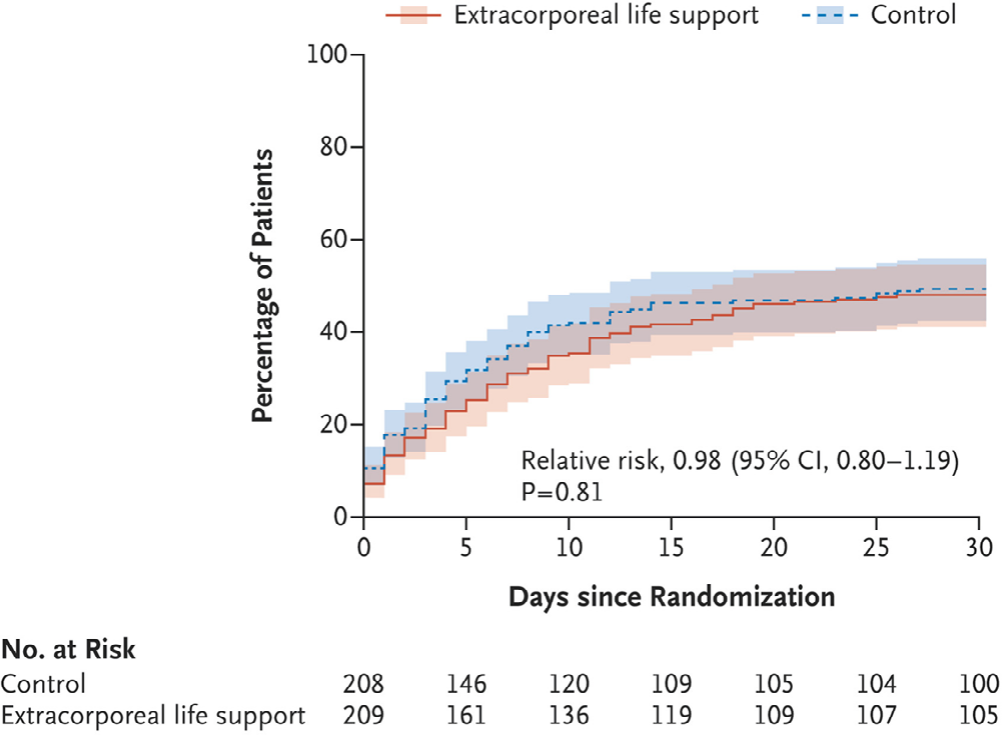
Primary endpoint death from any cause at 30 days in the Extracorporeal Life Support in Infarct‐related Cardiogenic Shock (ECLS‐SHOCK) trial.^5^ The shaded areas indicate the 95% confidence intervals.

It is important to stress that the ECLS‐SHOCK trial studied the routine use of VA‐ECMO in infarct‐related cardiogenic shock. As such, the study enrolled a relatively broad spectrum of patients with infarct‐related cardiogenic shock, across different stages of shock severity, including those with resuscitated cardiac arrest or older patients up to 80 years of age. However, it is not a trial in patients with refractory cardiac arrest and ongoing resuscitation where VA‐ECMO is also being studied and applied in clinical practice (termed extracorporeal cardiopulmonary resuscitation). Further, patients with cardiogenic shock due to mechanical complications of acute myocardial infarction (e.g. papillary muscle rupture or ventricular septal defect) were excluded. The results are also not applicable to non‐infarct‐related causes of cardiogenic shock or other shock forms in general.

Some technical aspects also deserve mentioning. The timing of VA‐ECMO initiation has been a matter of debate. In the ECLS‐SHOCK trial, VA‐ECMO was started in the cath lab soon after hospital admission in almost all patients. It is unclear whether there is any clinical benefit to a watch‐and‐wait strategy and to first monitoring the clinical course after coronary revascularization before deciding for or against VA‐ECMO.

At 5.8%, the rate of left ventricular (LV) unloading in the ECLS‐SHOCK trial was quite low. Unloading refers to the concept of reducing LV afterload which is usually increased in peripheral cannulation of VA‐ECMO as a consequence of the retrograde return flow into the abdominal aorta. Several percutaneous and surgical unloading strategies have been developed, all of which display positive hemodynamic effects yet also increase invasiveness and possibly complications. It is therefore at present not clear whether there is an overall clinical benefit of LV unloading. The first relatively small randomized has recently been published showing neutral results.^6^

## 1 IS THERE STILL A ROLE OF ECMO IN THE TREATMENT OF INFARCT‐RELATED CARDIOGENIC SHOCK?

For the majority of patients with infarct‐related cardiogenic shock early VA‐ECMO should be avoided if the goal is to improve mortality. However, most clinicians would agree that there are some patients with infarct‐related cardiogenic shock in whom VA‐ECMO can be a lifesaving procedure. The difficulty lies in characterizing these patients. On the one end of the spectrum, there are patients who will pass away with or without ECMO treatment, for example, due to causes largely unrelated to the hemodynamic or respiratory situation. On the other end of the spectrum, there will also be patients who will survive regardless of the intervention. This leaves us with only a subset of patients in whom a survival benefit might at least theoretically be possible. Even in this subfraction of patients, VA‐ECMO‐related complications could neutralize any positive effect.

In summary, the results of the ECLS‐SHOCK trial call for a very conservative approach regarding routine unselected early VA‐ECMO in patients with infarct‐relating cardiogenic shock. Future studies should focus on identifying if there is a (likely small) patient population which could possibly still benefit from this treatment. In addition, randomized trials are also needed for cardiogenic shock of causes other than acute myocardial infarction.

## References

[1] Thiele H , Zeymer U , Neumann F‐J , et al. Intraaortic balloon support for myocardial infarction with cardiogenic shock. N Engl J Med. 2012;367(14):1287‐1296. 10.1056/NEJMoa1208410 22920912

[2] Hochman JS , Sleeper LA , Webb JG , et al. Early revascularization and long‐term survival in cardiogenic shock complicating acute myocardial infarction. JAMA. 2006;295:2511‐2515. 16757723 10.1001/jama.295.21.2511 PMC1782030

[3] Hochman JS , Sleeper LA , Webb JG , et al. Early revascularization in acute myocardial infarction complicated by cardiogenic shock. SHOCK Investigators. Should we emergently revascularize occluded coronaries for cardiogenic shock? N Engl J Med. 1999;341(9):625‐634. 10460813 10.1056/NEJM199908263410901

[4] Becher PM , Schrage B , Sinning CR , et al. Venoarterial extracorporeal membrane oxygenation for cardiopulmonary support. Circulation. 2018;138(20):2298‐2300. 10.1161/circulationaha.118.036691 30571518

[5] Thiele H , Zeymer U , Akin I , et al. Extracorporeal life support in infarct‐related cardiogenic shock. N Engl J Med. 2023;389(14):1286‐1297. 10.1056/NEJMoa2307227 37634145

[6] Chul Kim M , Lim Y , Hun Lee S , et al. Early left ventricular unloading or conventional approach after venoarterial extracorporeal membrane oxygenation: The EARLY‐UNLOAD randomized clinical trial. *Circulation*. Printed online: October 18, 2023. doi: 10.1161/circulationaha.123.066179 37850383

